# Supplementary material for: Effects of individual variation and seasonal vaccination on disease risks
Source: Nat Commun. 2025 Sep 26;16:8471. doi: 10.1038/s41467-025-63375-5 (PMC12475144; doi:10.1038/s41467-025-63375-5)
Supplement: Supplementary file 1 — Supplementary Information [file 41467_2025_63375_MOESM1_ESM.pdf]

Supplementary Information for *Effects of individual variation and seasonal vaccination on disease risks*

WS Hart, J Amin, H Park, K Kitagawa, YD Jeong, AR Kaye, S Iwami, RN Thompson

Supplementary Methods

Analytical outbreak risk derivation

We consider the generalised renewal equation transmission model outlined in the Methods section of the main text, and define the following additional quantities:

- $g(\alpha)$ is the probability density function of the distribution of possible individual infectiousness factors. For generality, we initially derive an equation satisfied by the outbreak risk for an arbitrary distribution (with mean value one), before considering a gamma distribution with shape (dispersion) parameter k as assumed in the main text.
- $q_{t,\tau}(\alpha)$ is the extinction probability (one minus the outbreak risk) if a single infected individual with infectiousness factor α, who was infected on calendar day t, is introduced into the population at (the start of) day since infection τ (i.e., on calendar day $(t+\tau)$), assuming there are no other infected individuals at the time of introduction (and no further external pathogen introductions into the population).
- $q_t = \int_0^\infty q_{t,1}(\alpha)g(\alpha)\mathrm{d}\alpha$ is the overall extinction probability (averaged over the distribution of possible infectiousness factors) following the introduction one day post infection (note that we assume transmission cannot occur on the day

of infection, i.e., $w_0 = 0$) of an individual infected on day t. The outbreak risk is then $p_t = 1 - q_t$.

To derive an expression for $q_{t,\tau}(\alpha)$ (and therefore q_t), we condition on the number of transmissions generated by the first infected individual on the day of introduction, which follows a Poisson distribution with mean $\alpha R_{t+\tau} w_\tau$, to obtain

$$q_{t,\tau}(\alpha) = \sum_{j=0}^{\infty} \frac{(\alpha R_{t+\tau} w_\tau)^j e^{-\alpha R_{t+\tau} w_\tau}}{j!} q_{t,\tau+1}(\alpha) q_{t+\tau}^j. \quad \text{(S1)}$$

Here, $q_{t,\tau+1}(\alpha) q_{t+\tau}^j$ gives the extinction probability conditional on j transmissions occurring (since infection lineages are assumed to be independent). This sum can be evaluated as

$$\begin{aligned} q_{t,\tau}(\alpha) &= q_{t,\tau+1}(\alpha) e^{-\alpha R_{t+\tau} w_\tau} \sum_{j=0}^{\infty} \frac{(\alpha R_{t+\tau} w_\tau q_{t+\tau})^j}{j!} \\ &= q_{t,\tau+1}(\alpha) e^{-\alpha R_{t+\tau} w_\tau (1 - q_{t+\tau})}. \quad \text{(S2)} \end{aligned}$$

Therefore, assuming $q_{t,\infty}(\alpha) = 1$, we have

$$\begin{aligned} q_{t,1}(\alpha) &= q_{t,2}(\alpha) e^{-\alpha R_{t+1} w_1 (1 - q_{t+1})} \\ &= \cdots = q_{t,\infty}(\alpha) \prod_{\tau=1}^{\infty} e^{-\alpha R_{t+\tau} w_\tau (1 - q_{t+\tau})} \\ &= \exp\left(-\alpha \sum_{\tau=1}^{\infty} R_{t+\tau} w_\tau (1 - q_{t+\tau}) \right). \quad \text{(S3)} \end{aligned}$$

Finally, we have

$$q_t = \int_0^{\infty} q_{t,1}(\alpha) g(\alpha) \mathrm{d}\alpha = \int_0^{\infty} \exp\left(-\alpha \sum_{\tau=1}^{\infty} R_{t+\tau} w_\tau (1 - q_{t+\tau}) \right) g(\alpha) \mathrm{d}\alpha. \quad \text{(S4)}$$

Therefore, the outbreak risk, $p_t = 1 - q_t$, satisfies

$$p_t = 1 - \int_0^\infty \exp\left(-\alpha \sum_{\tau=1}^{\infty} R_{t+\tau} w_\tau p_{t+\tau}\right) g(\alpha) \mathrm{d}\alpha. \qquad \text{(S5)}$$

In the specific case where the infectiousness factors, α, follow a gamma distribution with shape parameter k and (to ensure a mean value of one) scale parameter $1/k$, we then have

$$\begin{aligned} p_t &= 1 - \int_0^\infty \frac{k^k}{\Gamma(k)} \alpha^{k-1} \times \exp\left(-\alpha\left(k + \sum_{\tau=1}^{\infty} R_{t+\tau} w_\tau p_{t+\tau}\right)\right) \mathrm{d}\alpha \\ &= 1 - \left(1 + \frac{1}{k}\sum_{\tau=1}^{\infty} R_{t+\tau} w_\tau p_{t+\tau}\right)^{-k}, \end{aligned} \qquad \text{(S6)}$$

where the second equality follows by noting that the integrand in the first expression is proportional to the probability density function of a gamma distribution with shape parameter k and scale parameter $(k + \sum_{\tau=1}^{\infty} R_{t+\tau} w_\tau p_{t+\tau})^{-1}$.

Outbreak risk with periodic transmissibility

We now suppose that the instantaneous reproduction number, R_t is a periodic function of time with period T (i.e., $R_{t+T} = R_t$), so that the outbreak risk, p_t, must also be periodic with the same period. For $1 \le t \le T$, equation S6 then gives

$$\begin{aligned} p_t &= 1 - \left(1 + \frac{1}{k}\sum_{j=0}^{\infty}\sum_{s=1}^{T} R_{t+jT+s} w_{jT+s} p_{t+jT+s}\right)^{-k} \\ &= 1 - \left(1 + \frac{1}{k}\sum_{s=1}^{T} R_{t+s} v_s p_{t+s}\right)^{-k}, \end{aligned} \qquad \text{(S7)}$$

where $v_s = \sum_{j=0}^{\infty} w_{jT+s}$. Note that if the generation time is always shorter than the period, T, then we simply have $v_s = w_s$. Now, equation S7 can be re-written as

$$p_t = 1 - \left(1 + \frac{1}{k}\sum_{s=1}^{T} B_{t,s} p_s\right)^{-k}, \tag{S8}$$

where $B_{t,s}$ is the $(t,s)^{\text{th}}$ entry of the $T \times T$ matrix,

$$B = \begin{pmatrix} \nu_T R_1 & \nu_1 R_2 & \cdots & \nu_{T-1} R_T \\ \nu_{T-1} R_1 & \nu_T R_2 & \cdots & \nu_{T-2} R_T \\ \vdots & \vdots & \ddots & \vdots \\ \nu_2 R_1 & \nu_3 R_2 & \cdots & \nu_1 R_T \\ \nu_1 R_1 & \nu_2 R_2 & \cdots & \nu_T R_T \end{pmatrix} \tag{S9}$$

Equation S8 gives a closed system of T equations in T variables $(p_1, \dots, p_T)$, which can be solved numerically. While equation S8 always has a solution with each p_t equal to zero, in all our numerical examples we obtained a (numerical) solution with each p_t strictly between zero and one, which was assumed to give the true outbreak risk values (this can be verified using model simulations, as in Fig. 1E of the main text).

Calculation of COVID-19 susceptibility profile

Here, we provide details of the calculation of the expected susceptibility, $\bar{S}(\tau)$, of an individual included in annual vaccination campaigns, as a function of time since most recent vaccine dose, τ.

We considered a large synthetic cohort of $n = 10{,}000$ individuals, labelled $i = 1, \dots, n$. For each individual, we sampled each antibody dynamics model parameter, $\theta_i \in \{H_i, m_i, K_i, \mu_i, \tau_{d,i}, D_i, \delta_i\}$ as $\theta_i = \theta_{pop} \exp(\omega_\theta \varepsilon_{\theta,i})$, where θ_{pop} and ω_θ are the assumed population (median) parameter and standard deviation of random effect, respectively (as listed in Supplementary Table 1), and $\varepsilon_{\theta,i}$ is a random variate drawn from a normal distribution with mean zero and standard deviation one (independently for different parameters and individuals). The individual parameter values were then

used to generate an individual antibody titre profile, $A_i(\tau)$, given by the periodic solution of the antibody dynamics model (equations 6-7 in the main text) with period $T = 365$ days. This periodic solution was generated by numerically solving equations 6-7 on $\tau \geq -5T$ with boundary condition $A_i(-5T) = 0$, and with vaccination assumed to occur at each time jT for integer $j \geq -5$, and then taking the resulting solution for $\tau \in [0, T)$. Equation 8 in the main text was then used to obtain the individual susceptibility profile, $S_i(\tau)$. We then calculated the expected susceptibility,

$$\bar{S}(\tau) = \frac{1}{n}\sum_{i=1}^{n} S_i(\tau). \tag{S10}$$

Additionally, we calculated 95% prediction intervals for individual antibody titres and susceptibility levels at each time τ (although these were not used in outbreak risk calculations).

<u>**Supplementary Figures**</u>

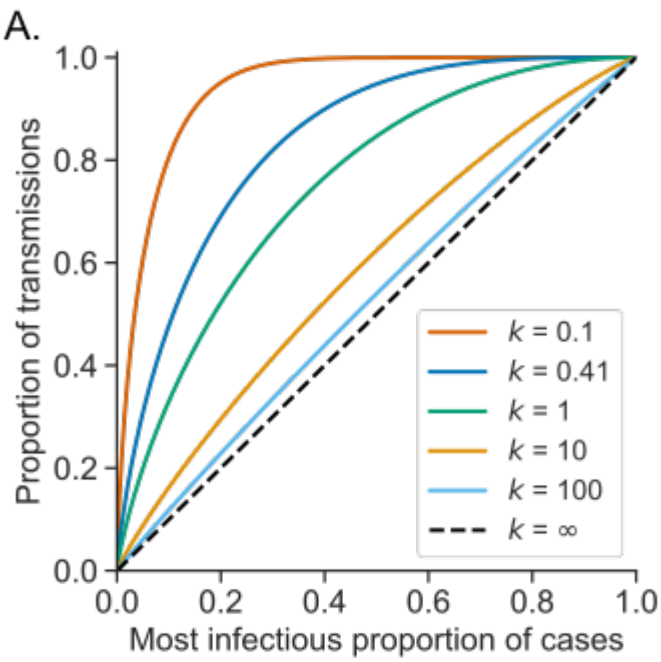

Supplementary Figure 1. Heterogeneity in transmission between individuals. For each specified proportion of infected individuals, ordered from the most to the least infectious (based on an assigned individual infectiousness factor – see Fig. 1B), the proportion of expected total transmissions generated by those individuals is plotted for a range of values of the dispersion parameter, k (the shape parameter of the gamma distribution of infectiousness factors; $k = 0.1$: red; $k = 0.41$: blue; $k = 1$: green; $k = 10$: orange; $k = 100$: light blue; $k = \infty$: black dashed). A greater extent of heterogeneity (i.e., degree of superspreading) is seen for smaller values of k, since a larger proportion of transmissions are then generated by a given (ordered) proportion of infected individuals. For details of the calculation underlying this figure, see (1).

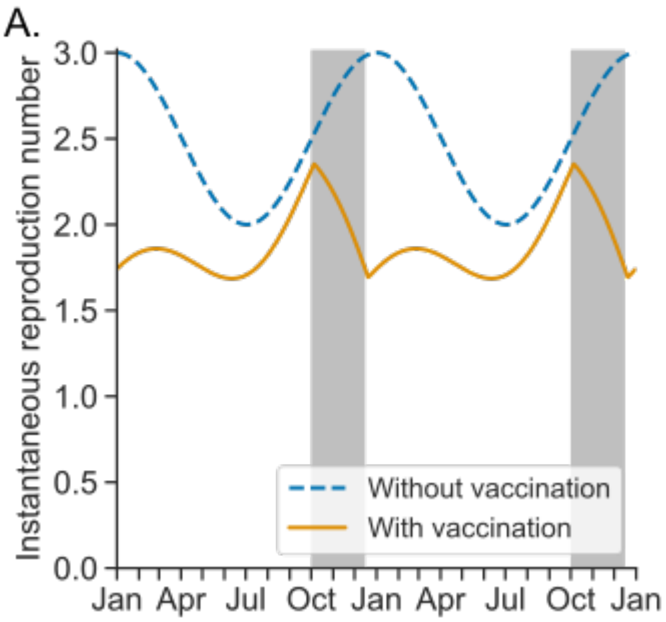

Supplementary Figure 2. Reproduction number under booster vaccination. The instantaneous reproduction number, R_t, both without booster vaccination ($R_t = R_{0,t}$; blue dashed curve), and when booster vaccination takes place ($R_t = \eta_t R_{0,t}$; orange curve), is shown under the default model inputs considered in Fig. 2.

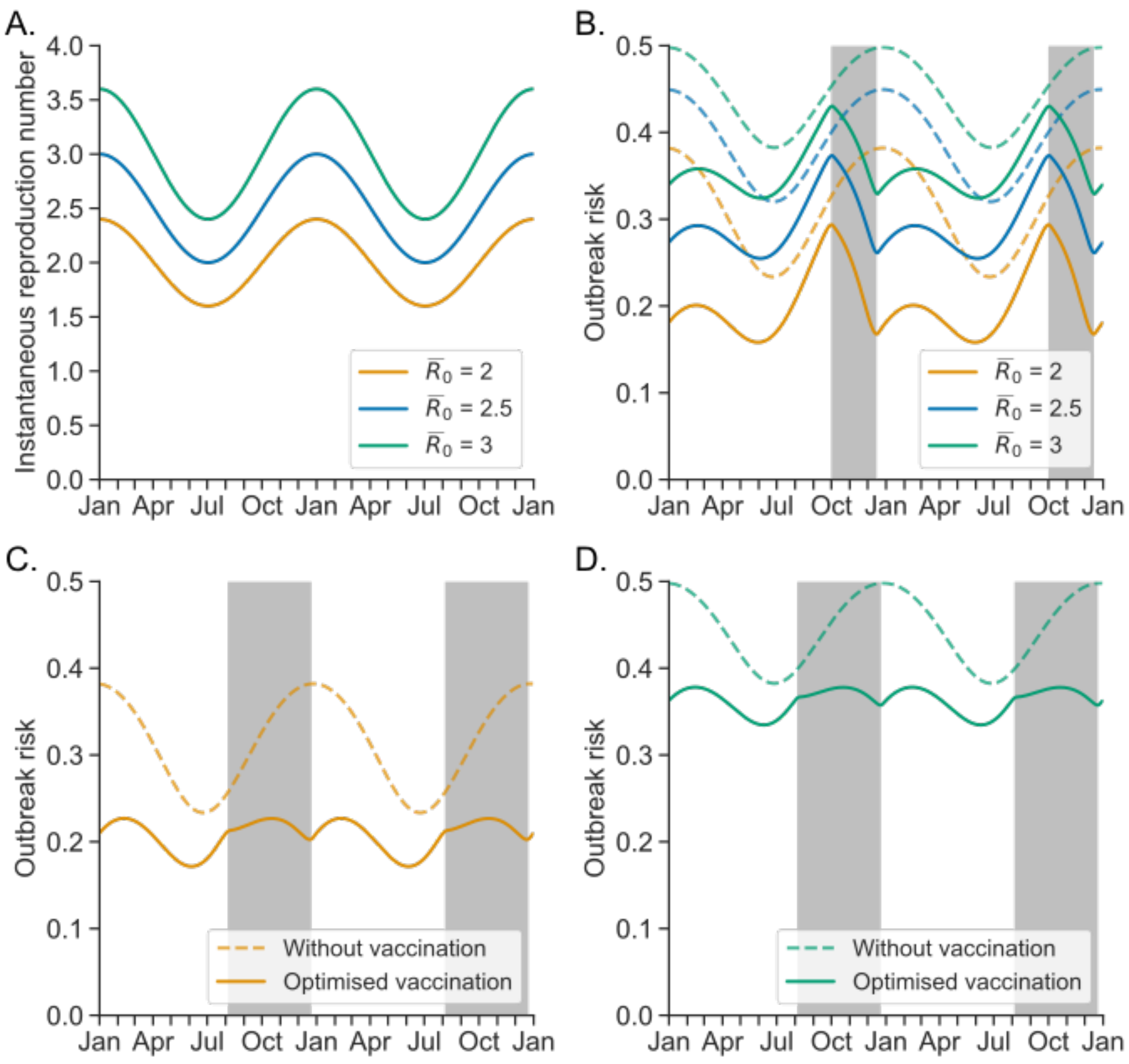

Supplementary Figure 3. Sensitivity of results to the annual mean reproduction number in the absence of booster vaccination, $\overline{R_0}$. A. Instantaneous reproduction number for values of $\overline{R_0} = 2$ (orange curve), 2.5 (our default assumed value; blue curve) and 3 (green curve). B. Outbreak risk without booster vaccination (dashed curves) and under the default assumed timing of booster vaccine distribution (solid curves) for the three values of $\overline{R_0}$ (same colour key as in A). C-D. Outbreak risk without booster vaccination (dashed curves) and with optimised booster vaccine distribution timing (solid curves; the optimal time period each year is shown in the grey shaded regions) for $\overline{R_0} = 2$ (C) and $\overline{R_0} = 3$ (D).

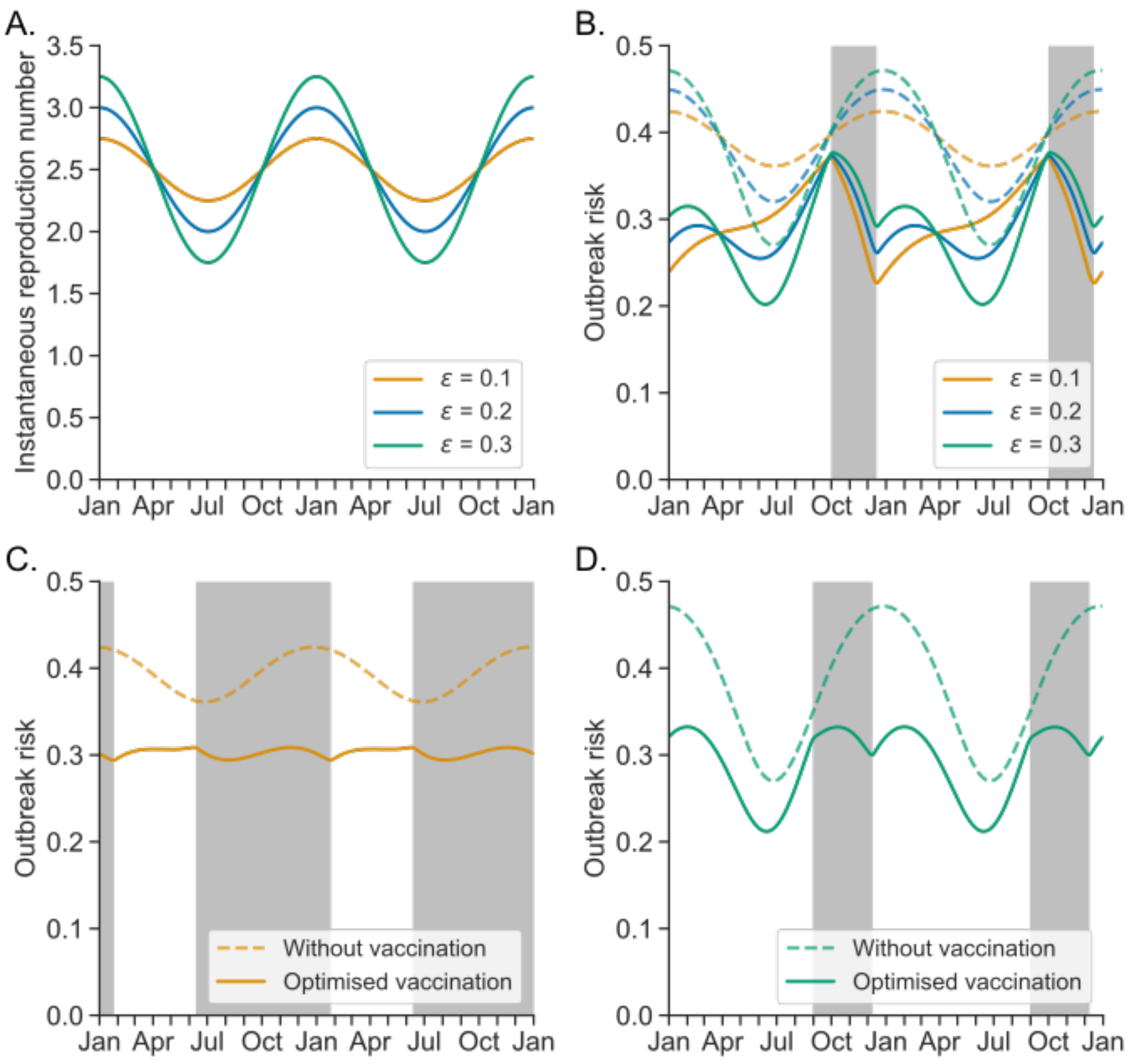

Supplementary Figure 4. Sensitivity of results to the extent of seasonality of transmission in the absence of booster vaccination, ε. The value of ε gives the proportion by which the annual maximum and minimum instantaneous reproduction number each differ from the annual mean value (see equation 10 in the main text). A. Instantaneous reproduction number for values of $\varepsilon = 0.1$ (orange curve), 0.2 (our default assumed value; blue curve) and 0.3 (green curve). B. Outbreak risk without booster vaccination (dashed curve) and under the default assumed timing of booster vaccine distribution (solid curves) for the three values of ε (same colour key as in A). C-D. Outbreak risk without booster vaccination (dashed curves) and with optimised booster vaccine distribution timing (solid curves; the optimal time period each year is shown in the grey shaded regions) for $\varepsilon = 0.1$ (C) and $\varepsilon = 0.3$ (D).

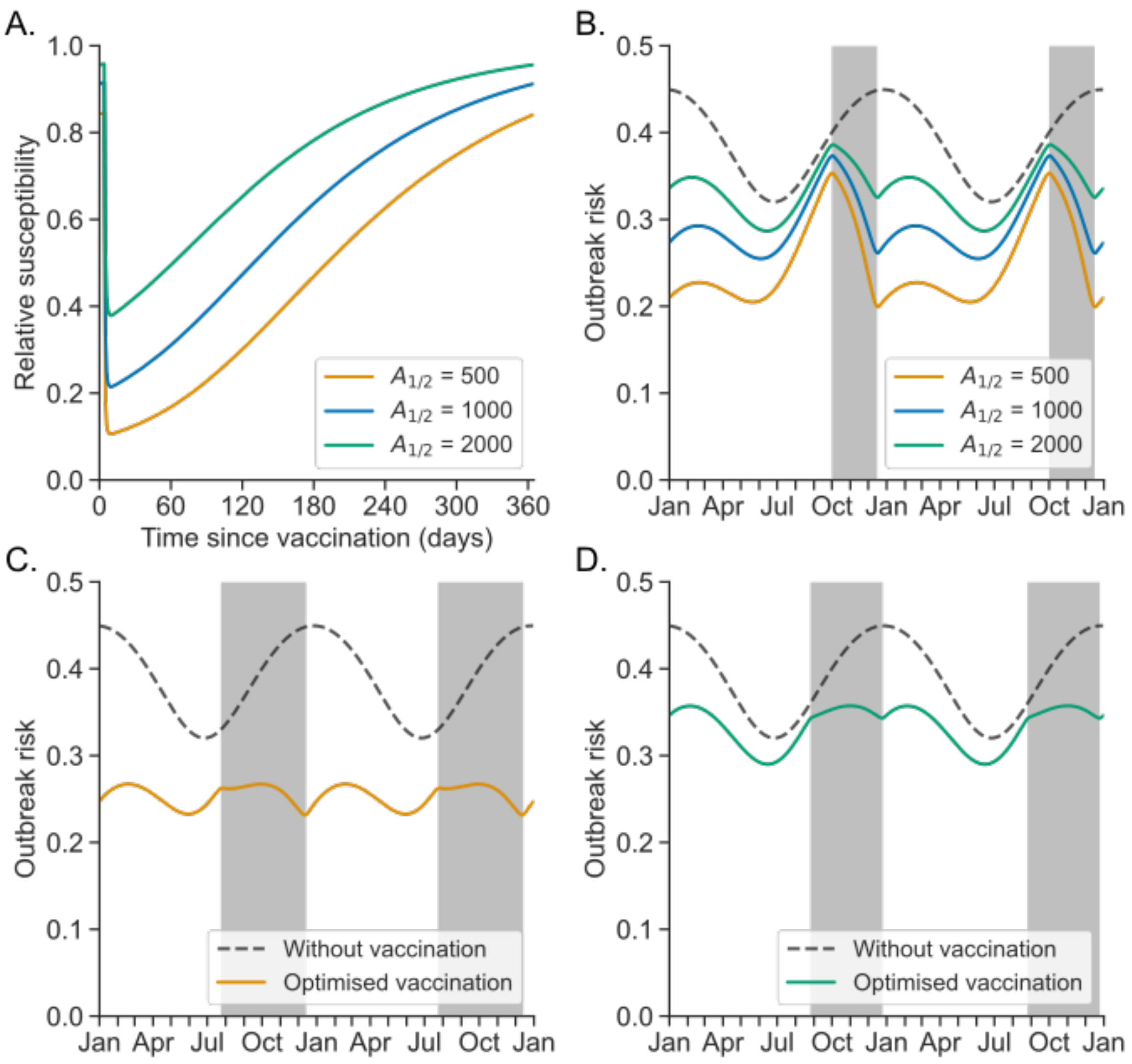

Supplementary Figure 5. Sensitivity of results to the extent of vaccine protection. The extent of vaccine protection is characterised by the parameter $A_{1/2}$, which gives the antibody titre at which 50% protection against infection is conferred (see equation 8 in the main text), so that a higher value of $A_{1/2}$ corresponds to a smaller extent of vaccine protection (i.e., higher susceptibility to infection). A. Average relative susceptibility to infection (calculated over a synthetic cohort of 10,000 individuals) as a function of time since (most recent) vaccination for $A_{1/2} = 500$ (orange curve), $1{,}000$ (our default assumed value; blue curve) and $2{,}000$ AU/mL (green curve). B. Outbreak risk without booster vaccination (black dashed curve) and under the default assumed timing of booster vaccine distribution (solid curves) for the three values of $A_{1/2}$ (same colour key as in A). C-D. Outbreak risk without booster vaccination (black dashed curves) and with optimised booster vaccine distribution timing (solid curves; the optimal time period each year is shown in the grey shaded regions) for $A_{1/2} = 500$ (C) and $A_{1/2} = 2{,}000$ (D).

Supplementary Table

Parameter	Description	Population value	Standard deviation of random effect
H	Maximum antibody production rate	6,090 AU/mL	0.697
m	Steepness parameter of antibody production response to mRNA	0.0176	0.369
K	mRNA amount at which antibody production rate takes half its maximum value	28,100 μg/0.5mL	0
μ	Antibody decay rate	0.875 day^{-1}	0
τ_d	Delay before induction of antibody response	4.09 days	0
D	mRNA dose	100 μg/0.5mL	0
δ	mRNA decay rate	0.693 day^{-1}	0

Supplementary Table 1. Population values and random effect parameters for individual antibody model parameters. We assumed individual values of each parameter, $\theta \in \{H, m, K, \mu, \tau_d, D, \delta\}$, of the antibody dynamics model (equations 6-7 in the main text) to be distributed as $\theta = \theta_{pop} \exp(\omega_\theta \varepsilon_\theta)$, where θ_{pop} is the population median value, ω_θ is the standard deviation of the random effect, and ε_θ is a random variate drawn from a normal distribution with mean zero and standard deviation one. For each such parameter, θ, the values of $\log(\theta_{pop})$ and ω_θ were taken to be the mean and standard deviation between individuals, respectively, of the natural logarithm of the individual parameter estimates obtained in (2). Specifically, in (2), individual estimates of the parameters H and m were obtained by fitting the antibody dynamics model to longitudinal data collected from 1,618 individuals following booster vaccination. The parameters K, μ and τ_d were assumed to take the specified fixed values in (2) based on separate parameter estimates obtained in that study using more densely sampled data from 12 healthcare workers (with this estimation indicating limited variability in these three parameters (2)), while the values of D and δ were assumed in that study (and we take the same values here).

Supplementary References

1. Lloyd-Smith, J. O., Schreiber, S. J., Kopp, P. E. & Getz, W. M. Superspreading and the effect of individual variation on disease emergence. *Nature* **438,** 355–359 (2005).
2. Park, H. *et al.* Longitudinal analysis of antibody titers after primary and booster mRNA COVID-19 vaccination can identify individuals at risk for breakthrough infection. Preprint at https://www.biorxiv.org/content/10.1101/2025.03.01.639360v1 (2025).
